# Tolerance and immunity to pathogens in early life: insights from HBV infection

**DOI:** 10.1007/s00281-017-0641-1

**Published:** 2017-07-06

**Authors:** Michelle Hong, Antonio Bertoletti

**Affiliations:** 1 Emerging Infectious Diseases (EID) Program, Duke-NUS Medical School, 8 College Road, Singapore, 169857 Singapore; 2 Viral Hepatitis Laboratory, Singapore Institute for Clinical Sciences, Agency of Science Technology and Research (A*STAR), Singapore, Singapore

**Keywords:** Hepatitis B virus, Vertical transmission, Immune tolerance, HBV-specific T cells, Liver inflammation, Trained immunity

## Abstract

Immunity is not static but varies with age. The immune system of a newborn infant is not “defective” or “immature.” Rather, there are distinct features of innate and adaptive immunity from fetal life to adulthood, which may alter the susceptibility of newborn infants to infections compared to adults. Increased protection to certain infectious diseases during early life may benefit from a dampened immune response as a result of decreased immune pathology. This concept may offer an alternative interpretation of the different pathological manifestations clinically observed in hepatitis B virus (HBV)-infected patients during the natural history of infection. Herein, we review the immune pathological features of HBV infection from early life to adulthood and challenge the concept of a generic immune tolerant state in young people. We then discuss how the different clinical and virological manifestations during HBV infection may be related to the differential antiviral immunity and pro-inflammatory capacity generated at different ages. Lastly, we address the potential to consider earlier therapeutic intervention in HBV-infected young patients to achieve effective immune control leading to better outcomes.

## HBV viral transmission and disease burden

Mother-to-child transmission of viruses (transplacental, perinatal, or postnatal) is often associated with higher levels of viral replication, a greater risk of persistent or chronic infection, and more severe disease outcome compared to those acquired during adulthood [1]. Hepatitis B virus (HBV), among the intracellular pathogens which can be acquired through mother-to-child transmission, causes one of the most common infectious diseases in the world.

HBV is a hepatotropic, non-cytopathic, DNA virus that chronically infects approximately 240 million people worldwide [2, 3], with liver-related morbidity and mortality due to chronic hepatitis B (CHB) including cirrhosis and hepatocellular carcinoma accounting for more than 600,000 deaths per year [4]. HBV infection causes acute or chronic liver diseases characterized by different levels of liver inflammation and viral replication, which in turn, is determined by a complex interplay of host and viral factors including host genetic background, dose or route of infection, viral genotype, and importantly, the age of acquisition [5, 6]. The risk of developing chronic infection is the highest following vertical or perinatal infection (90%) compared to those infected between the ages of 1 and 5 years (20–30%) or during adulthood (<5%) [7, 8]. In regions of high endemicity, including Asia and sub-Saharan Africa, virus transmission from mother-to-child at birth is the major cause of HBV chronicity. Despite the development of an effective HBV prophylactic vaccine [9, 10], the prevalence of the virus continues to increase [3].

## Triggering immunity and tolerance to HBV

**Fig. 1:**
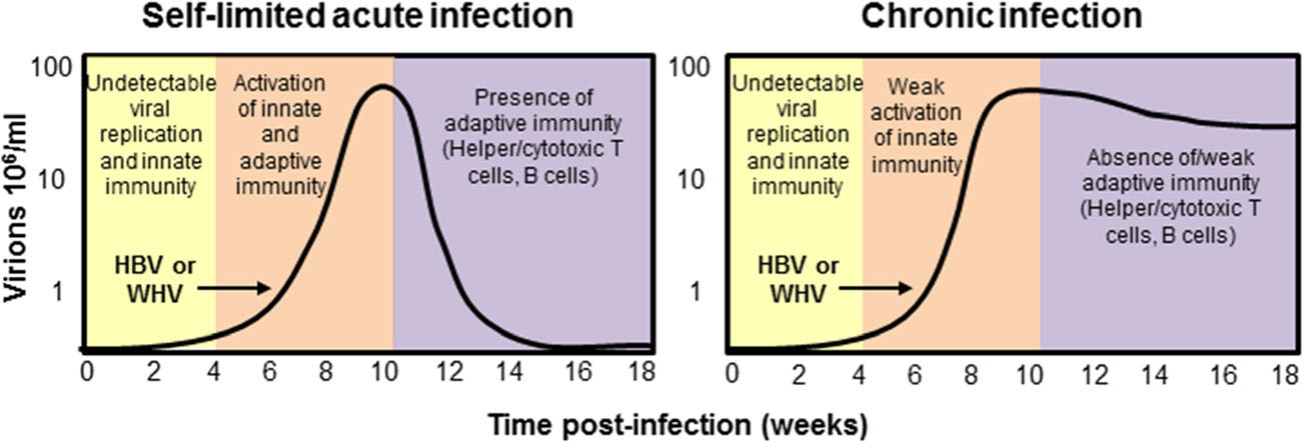
Kinetics of HBV replication and host immune response in self-limited versus chronic infection. Coordinated activation of both innate and adaptive immunity is necessary for successful control of HBV. *HBV* hepatitis B virus, *WHV* woodchuck hepatitis virus

HBV infection, unlike many other viruses, is characterized by a delayed kinetics of viral replication and spread. Experimental data collected, mainly in animal models but also in humans [11], showed that HBV replication is detectable in the serum or the liver of infected host only 4–7-week post-infection [11–16] (Fig. 1). Following this period, HBV enters a logarithmic phase of expansion, reaching 10^9^–10^10^ copies/ml in the liver and serum [17], and infecting most hepatocytes [11, 13–16] (Fig. 1). A further unique characteristic of HBV is its inability to trigger a classical innate immune response. Data in vitro and in vivo has shown the absence of activation of type I IFN genes during the logarithmic phase of HBV expansion and the absence of pro-inflammatory cytokines in the serum of patients in the early phases of acute infection. The causes of this inability of HBV to activate a classical innate immune response and whether HBV actively suppress innate immunity or only evade its recognition have been highly debated and have been reviewed elsewhere [18].

HBV is, however, able to trigger a functional and efficient adaptive immune response characterized by the presence of HBV-specific antibodies and T cell response in most of the patients infected horizontally. In this case, T cell response develops before the peak of acute hepatitis and is preceded by the production of IFN-γ within the hepatic parenchyma, which has been suggested to indicate a possible natural killer (NK) or NK-T cell activation. Such induction of a robust and multi-specific antiviral T cell response is necessary for the control of the infection, and deletion of CD8^+^ T cells in acutely infected chimpanzees demonstrates the pivotal role of these components of the immune system in HBV control.

The majority of infections acquired at birth or perinatally develops instead into chronic infection that are characterized by a profound defect of HBV-specific T cell response, with lower numbers of circulating and intrahepatic HBV-specific CD8^+^ and CD4^+^ T cells, as well as lower and restricted production of HBV-specific antibody [19] (Fig. 1). The reason why the virus is unable to trigger an efficient antiviral immunity under such circumstances is open to debate. It is interesting to point out that neonatally infected woodchucks that develop chronicity lack the IFN-γ and TNF-α surge [20–22] which is present in animals that are able to control the infection, and fail to develop an efficient antiviral specific immune response. Presentation of HBV antigens by hepatocytes in the absence of pro-inflammatory response may induce a defective T cell response that is further depleted by prolonged exposure to large quantities of soluble HBV antigens (HBeAg and HBsAg) [23].

HBeAg, a secretory form of the nucleocapsid antigen or core antigen, is produced in large excess during HBV replication [24]. The tolerizing effect of HBeAg has been well characterized in mice [25–27] and likely contributes to the low level of core-specific T cell responses present in HBeAg^+^ chronic patients. Soluble form of HBsAg is also produced in excessive amounts during HBV replication. HBV particles composed of only HBsAg are produced up to 10^3^–10^6^-fold excess over whole virions and can reach 1–10 μg/ml in the serum [24]. Such persistently high viral antigen load allows cross-presentation by liver professional antigen-presenting cells (Kupffer cells, liver endothelial cells), which can induce tolerance in chronic HBV patients [28]. Furthermore, direct presentation of foreign antigen by hepatocytes has been suggested to preferentially induce CD8^+^ T cell tolerance, resulting in reduced CD8^+^ T cell clonal expansion and increased T cell apoptosis [28]. Other factors, such as dendritic cell functional alteration and T regulatory cells (Tregs), have been suggested to contribute to virus-specific T cell tolerance in chronic HBV patients. However, the evidence supporting the role of dendritic cells or Tregs in the induction of antigen-specific tolerance remains controversial [29].

## Does vertical HBV transmission induce immune tolerance?

Chronic HBV infection, particularly in Asia, is caused by mother-to-child transmission of the virus. HBV infection in infants or young children is usually asymptomatic until late adulthood, when it causes liver pathologies (cirrhosis and hepatocellular carcinoma) [3]. To explain this dichotomy, HBV is thought to “hijack” the immaturity of the neonatal immune system and/or to induce an “immunotolerant phase” of disease—characterized by high HBV DNA levels, positive hepatitis B e antigen (HBeAg), normal or low serum alanine aminotransferase (ALT) levels, and minimal or no liver inflammation that usually lasts from a few years to several decades without disease progression [5, 7, 30].

This immunotolerance hypothesis is mainly supported by data from experimental animal models (i.e., HBV transgenic animals) that showed the presence of immunological defects which impair HBV-specific T and B cell priming in neonatal animals [27, 31, 32], thus predisposing to HBV chronicity. A recent study by Tian et al. [33] showed that the qualitative and quantitative defects detected in HBV-specific CD8^+^ T cells in vertically infected neonatal mice were associated with inhibitory responses exerted by hepatic macrophages, leading to increased risk of vertical transmission and development of chronicity. While these data are methodologically robust, their significance in relation to HBV pathogenesis remains questionable, since the data supporting such immunological features are derived exclusively from HBV transgenic animals that do not support natural HBV infection. Instead, HBV virions are produced from HBV transgenes introduced into the mouse genome under the control of hepatocyte-specific promoter, and as such cannot fully recapitulate the natural course of HBV infection. Certainly, a lack of appropriate animal models represents a major hurdle to study immunotolerance in HBV [34].

The concept that the neonatal immune response is somehow “defective” or “immature” is also changing, and there is mounting evidence showing that the neonatal immune responses defy such simple categorization. In fact, neonates seem to embody the full spectrum of immune responses, including immune effectors and regulatory responses, during early life [35–37], and they are neither “immunodeficient” nor “immature.” Newborn infants have also been shown to harbor the ability to mount virus-specific T cell response towards viral infections in early life [38–40]. For example, Luzuriaga et al. demonstrated that HIV-1-specific T cells can be detected in some young infants, and even the fetus [38]. Studies of congenital CMV infection showed that high frequencies of ɣδ and αβ T cells can be induced early during fetal life [39, 40].

Furthermore, exposure of the newborn immune system towards microbes at birth can also shape the maturation status of the newborn infant. Epidemiological and experimental evidences have shown that exposure to bacterial or viral infections after birth and vaccination with live vaccines can protect infants against unrelated pathogens by inducing an increased functional efficiency of their innate immune system through a process known as “trained immunity” [41]. For example, BCG vaccination in newborn children in West Africa is associated with reduced all-cause mortality, largely due to infections other than tuberculosis, in the first month of life [42]. Similar observations have also been made for vaccination with vaccinia virus [43] or with measles vaccine [44], which showed important non-specific protective effects. Further evidence that trained immunity could be clinically relevant in vivo during early life is exemplified by the diminished risk of late-onset sepsis (i.e., after 72 h of life) among very premature infants with early-onset sepsis (i.e., within the first 72 h of life) [45]. All these earlier reports indicate that the immune system of newborn infants is not “immature” or “defective” per se, and that the innate immune system could display memory features right from birth. Furthermore, the newborn immune system appears to be less prone to trigger a full-blown pro-inflammatory reaction, likely as an evolutionary adaptation to prevent undesirable immune reactions in utero.

Therefore, the hypothesis that vertical HBV infection induces a state of general immunotolerance, the basis of which the disease is managed and treatment decisions are made, remains controversial. Although the immunological data both during and after natural vertical HBV infection is limited, several epidemiological and experimental evidences can be used to challenge this concept of immunotolerance. For example, the functionality of dendritic cells is intact or minimally altered in neonates of HBV^+^ mothers [46–48]. Furthermore, T cells specific for the HBV core and polymerase antigens (antigens not present in the HBV prophylactic vaccine) have been detected in HBsAg^−^ children born to HBV^+^ mothers in two independent studies [49, 50]. This shows that neonates born to HBV^+^ mothers do not necessarily harbor defects in T cell priming. On the contrary, they have the ability to prime HBV-specific T cell responses. Analysis of HBV quasispecies in children with a clinical profile labeled as immunotolerant showed a high HBV diversity [51], a virological profile that is compatible with the presence of an active immune pressure and not with complete immune tolerance during this initial phase of infection.

Besides, the efficacy of HBV vaccination at birth in children of HBV^+^ mothers [52, 53] raises doubts that the state of complete HBV immune tolerance and the broad defects in T and B cell interaction detected in murine models exist during natural infection. The dogma of immunotolerance in vertical HBV-infected children is also in contrast to epidemiological observations showing higher frequency of HBV-related fulminant hepatitis in infants <1 year of age compared to that in older subjects [54], and with the observations obtained from malaria-HBV co-infected young patients in whom reduced parasitemia [55] and increased incidences of cerebral malaria [56], a Th1-mediated malaria complication, have been reported. Such observations are more suggestive of the possibility of an alternative relationship between HBV and humans during early life, i.e., HBV may play more than just a pathogenic role in humans.

**Fig. 2:**
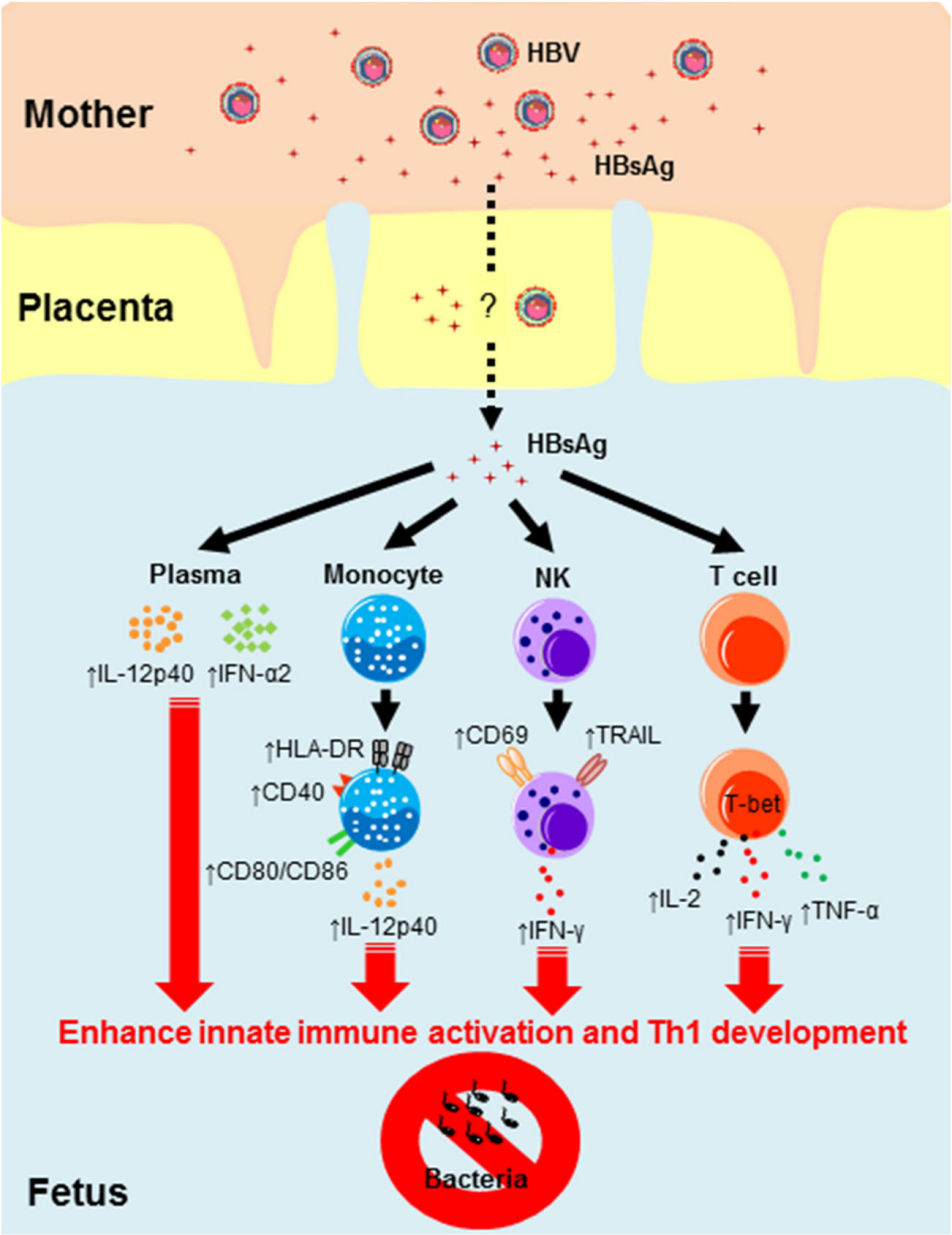
Induction of trained immunity in human neonates of HBV^+^ mothers. HBsAg^+^ cells can be detected in the cord blood of neonates born to HBV-infected mothers, demonstrating in utero exposure to viral products. These HBsAg^+^ cells could be due to transplacental passage of maternal HBsAg^+^ cells or active uptake of serum HBsAg by neonatal cells. HBV exposure in utero is associated with significantly elevated plasma levels of the antiviral cytokine IL-12p40, and in some cases, IFN-α2. Exposure to HBV in utero also induces innate immune cell (monocytes, NK cells) maturation/activation and enhances Th1 T cell development. Importantly, this heightened state of innate immune functionality results in a stronger ability of neonatal immune cells to respond to unrelated pathogen challenge—a process known as “trained immunity”

An indirect demonstration of this alternative relationship between HBV and humans comes from our recent work where we performed a detailed characterization of the immunological parameters in the cord blood of neonates born to HBV^+^ mothers. Contrary to the dogma of generic immunotolerance, we found that HBV exposure in utero triggers a state of “trained immunity,” characterized by increased innate immune cell activation and Th1 development, which in turn enhances the ability of HBV-exposed cord blood immune cells to respond to bacterial infection in vitro [57] (Fig. 2). These training effects are associated with alterations in the cytokine environment. Specifically, we detected, in the sera of HBV-exposed neonates, a cytokine signature compatible with a Th1-like response with higher production of IL-12p40 and in some cases IFN-α2, lower production of IL-10 and pro-inflammatory cytokines (such as IL-6, IL-8, and TNF-α). This Th1 cytokine signature is more suggestive of a symbiotic relationship between HBV and humans during early life, similar to that already demonstrated in murine models of herpesvirus-persistent infection [58], than to the induction of a tolerogenic response. HBV-specific T cells were not detected in the cord blood of HBV-exposed neonates; therefore, the possibility that HBV vertical infection might tolerize HBV-specific immunity cannot be excluded. Nevertheless, the induction of a trained immunity profile with a general Th1 response and suppression of pro-inflammatory events in HBV-exposed neonates show that the neonatal immune system can be “trained” by HBV exposure and further activated to possibly counteract unrelated pathogens during early life.

## Immunological and virological events during the “immunotolerant” phase of HBV infection

Most of the evidences supporting an immunotolerance disease phase of HBV infection during early childhood are based on clinical and virological parameters. HBV is not directly cytopathic, and HBV-specific CD8^+^ T cells control virus replication by recognizing and killing HBV-infected hepatocytes [16]. ALT is released from dying hepatocytes, and thus, serum ALT has been interpreted as a marker of immune activity, i.e., the presence or absence of ALT fluctuations correlates with the presence or absence of HBV-specific T cells. In this context, normal or minimal alterations in ALT levels, detectable in the majority of HBV-infected children, have been perceived as an indication of lack of HBV-specific T cell response. On the other hand, fluctuations in the levels of ALT and HBV DNA replication, more commonly observed during adulthood, is interpreted as an “awakening” of HBV-specific immunity.

Here lies a fundamental problem—the use of a clinical categorization to define a disease state in an immune-mediated liver condition is inappropriate and inaccurate. In reality, both experimental data in animal models and in humans during natural HBV infection have shown that ALT measurement cannot be used as a reliable surrogate of virus-specific T cell responses. Studies performed in adenovirus-infected mice have shown that T cell immunity against hepatocytes could occur without elevation in serum ALT level [59]. Furthermore, adoptive transfer of HBV-specific T cells can lead to significant inhibition of HBV replication without increase in serum ALT through cytokine-mediated non-cytopathic effect [60]. Direct quantification of HBV-specific T cells in the blood and liver of CHB patients have also shown that, in contrast to patients with acute hepatitis B [61], the quantity of HBV-specific T cells does not correlate with ALT levels [62, 63]. Instead, robust inflammatory events in the liver causing fluctuations in ALT levels, demonstrated both in adult mice and in patients, are associated with intrahepatic recruitment of granulocytes, monocytes, and non-antigen-specific T cells [62, 64, 65].

There is also mounting evidence showing that surrogate markers such as ALT might not reflect the true disease status, and what constitutes a normal or healthy ALT level continues to be debated. The new cut-off value recommended as healthy by Prati et al. [66], i.e., ≤30 U/l for men and ≤19 U/l for women, is higher than the traditional cut-off value of 40 U/l, but the upper limit of normal for children has not been established. Therefore, even a slight increase in serum ALT may reflect changes in immune activity and disease profile [67]. For instance, it has been reported that substantial fibrosis and necroinflammatory activity already exist in the liver biopsy of some patients in the “immunotolerant” phase with the traditional serum ALT cut-off value of ~40 U/l [68].

The concept of immune tolerance phase being a quiescent disease phase with an absence of virus-specific T cells and minimal changes in liver histology is now increasingly being challenged. Our recent study of chronic hepatitis B-infected children and young adults with a clinical and virological profile labeled as “immunotolerant” showed the existence of HBV-specific T cell responses that is less compromised than that observed in CHB-infected adult patients in the “immune clearance” phase [69]. A further demonstration that these patients do not display any generic state of immunotolerance was exemplified by the superior ability of circulating T cells from young IT patients with CHB to produce type 1 T-helper cytokines including TNF-α and IFN-ɣ, compared to age-matched healthy controls. On the other hand, the production of immunosuppressive cytokines including IL-10 and IL-4 was not elevated in these patients. Detailed analysis of the phenotype of T cells in these patients showed that young IT patients with CHB have a partially exhausted T cell profile, compared to adult patients with a more exhausted T cell profile (CD8^+^ T cells that are PD-1^+^ and CD127^lo^). This could explain why older patients have a less favorable response to therapy, and suggest that earlier therapeutic intervention may be more advantageous in young people who lack a fully exhausted T cell profile.

Similarly, new data from Mason et al. further strengthen the concept that ongoing disease activity already exists in the liver of CHB patients during the “immunotolerant” phase [70]. For instance, Mason et al. reported an unexpectedly high number of HBV DNA integration sites randomly distributed across the human chromosome in patients clinically labeled as “immunotolerant,” comparable to those observed in patients with HBeAg^+^ disease or those in the immune clearance phase. Viral DNA integration and the resulting genomic instability is associated with the risk of developing hepatocarcinogenesis, with 80% of HBV-related HCC demonstrating clonal integrated HBV sequences [71]. In addition to HBV DNA integration, clonal hepatocyte expansion was also detected in “immunotolerant” patients at an unexpectedly high rate [70]. Clonal expansion of hepatocytes, a risk factor for the development of HCC, probably occurs in response to hepatocyte turnover mediated by HBV-specific T cell killing of infected hepatocytes, since HBV-specific T cells were detected in the peripheral blood of these patients. Furthermore, the maximum hepatocyte clone size did not differ between the “immunotolerant” patients and those with HBeAg^+^ disease or in the immune clearance phase. All these findings demonstrate that promoters of oncogenesis exist in all phases of CHB infection, even in patients at the early stage of CHB infection traditionally considered “immunotolerant.”

Collectively, our findings, together with others in the field, do not support the notion that the initial “immunotolerant” phase is completely devoid of markers of disease progression or that there is a lack of immune response. Like in all patients with chronic HBV infection, adaptive immunity is compromised in this phase, but not more than in the others. Hence, this so-called immunotolerant phase, characteristically associated with infection acquired during early life, has now been defined as HBeAg^+^ chronic infection according to the recent EASL guidelines to better reflect the immunological events occurring during natural HBV infection. However, arguments against such new definitions have raised an interesting debate [72, 73].

## Age-related changes in immunity and inflammatory responses during viral infections

It is well described that there are clear age-related differences in response to vaccines between early life and adulthood, but whether similar differences exist during natural infection with pathogenic microorganisms remains poorly defined [37]. In the context of HBV infection, we have reviewed the clinical and experimental evidences showing that the initial “immunotolerant” phase of chronic HBV infection (now defined as HBeAg^+^ chronic infection) is no longer considered to be quiescent, as the detection of HBV-specific T cells and liver damage point to underlying disease and immune activity. As such, how can we explain the different virological and inflammatory profiles during the natural history of HBV infection?

A possible explanation is that there are developmental differences in the immune function between infants/young children and adults. This concept is well established for adaptive immunity, but increasingly, it is appreciated that the innate immune function also changes with age. While the expression of TLR is similar in early versus adult life, TLR-mediated production of cytokines follows a developmental pattern: the production of anti-inflammatory cytokines (e.g., IL-10) is high in pre-term infants, then progressively declines over the first year of life, but is higher in children compared to that in adults [74]. In contrast, the production of pro-inflammatory cytokines (e.g., IL-1β, TNF-α) gradually increases during early life [74] until it reaches a state of chronic low-grade systemic inflammation called “inflammaging” present in older subjects [75]. As a consequence, T cell responses also shift from a skewed Th2/Treg type response in newborns to a more Th1-type response in children and adults [76] with a progressive increase in effector memory T cell pools, which can respond efficiently to a cognate infection and are more sensitive to cytokine-mediated activation [77].

Whether the different virological and inflammatory patterns observed during the natural history of HBV infection could be explained by this age-related modulation in immune function remains to be investigated. Nevertheless, we propose that this early disease phase, characterized by high level of HBV replication and low incidence of liver inflammatory events, might be caused by the induction of an antiviral “non-inflammatory” immune response [9]. Specifically, the few and functionally impaired HBV-specific T cells may, similar to adults, try to contain the infection by cytokine-dependent control and killing of HBV-infected hepatocytes. However, such responses do not trigger non-specific recruitment of inflammatory cells to the liver, possibly due to the dampened pro-inflammatory responses and the limited pool of effector/memory T cells present in children.

The concept that pathological processes might be modulated by age is evident in other infections. Influenza virus infection, for example, can cause death due to increased inflammatory response in the lung, thus predisposing to bacterial infection in some but not all individuals. During the 1918 influenza pandemic, it has been shown that children had a much lower mortality rate than young adults despite experiencing a higher rate of clinical influenza [78]. This phenomenon has also been observed in other bacterial and viral infections [79]. Although the immunological mechanisms underlying these differential outcomes have not been experimentally demonstrated, the emerging picture appears to be reduced pro-inflammatory responses in children than that in adults. Interestingly, it has been demonstrated, albeit in a small-scale longitudinal study, that dramatic fluctuations in serum ALT levels occur in CHB patients in the age range of 20–25 years old [51], which is the exact age window where higher pro-inflammatory events have been observed in humans.

## Implications for future treatment strategies for chronic hepatitis B—earlier treatment for young patients?

Understanding the immune pathogenesis of infectious diseases in early life is required for the development of effective interventional strategies for enhancing neonatal immune responses to protect young children. Historically, the goals of therapy for CHB patients are the reduction of viremia and amelioration of hepatic dysfunction, with the hope of delaying progression to cirrhosis and the subsequent development of HCC [30]. While a “sterilizing cure” of HBV with the removal of cccDNA and integrated virus is difficult or impossible to achieve, a “functional cure” whereby patients achieve sustained suppression of HBV viremia, loss of HBsAg after a defined course of therapy, and reach a health status equivalent to a person who has recovered spontaneously from HBV infection, should at least be the goal of next wave of therapies [30].

Current guidelines from the international liver association recommend treatment for CHB patients only when they show signs of clinically active disease or development of fibrosis, typically after the age of 30 years old. However, symptoms of advanced disease often appear later in life, at a stage when little can be done to alter the disease course. This could explain the poor response rate to therapies observed in adult patients, thus highlighting the limitations of current practice and the underlying need to better define the optimal timing for treatment. At the present moment, whether young CHB patients in the “immunotolerant” disease phase are indicated for treatment remains debated. The oversimplistic view that HBV vertical or perinatal infection results in a phase of HBV-specific immune tolerance which is lost in adulthood is not supported by our increased understanding of the effect of age on the immune response, nor is it supported by our better insight into the immunological events triggered by HBV infection [7]. The arguments presented above challenge the concept of a generic immunotolerant state in young people and more importantly, raise questions about the premise on which treatment decisions are made.

Chronic hepatitis B infection in children or young people represents a therapeutic challenge to practitioners given the paucity of data regarding treatment in these groups of young patients [80]. Nevertheless, there are some, albeit limited, studies that show promising results. For instance, a small pilot study by Carey et al. [81] has shown beneficial responses in a proportion of “immunotolerant” children treated with a combination therapy of lamivudine and pegIFN-α. Such beneficial responses were associated with an increase in HBV-specific T cell proliferation, reduction in HBV DNA levels, and notable increase in HBsAg seroconversion, thus providing further support for the potential benefit of early treatment in CHB patients [81, 82]. Similar studies performed in CHB-infected children have demonstrated that IFN-α was well tolerated, and children less than 5 years of age may have an enhanced response to IFN-α [83]. Likewise, the efficacy of tenofovir disoproxil fumarate was comparable between CHB-infected adolescents (<18 years old) with that observed in adult subjects [84]. Nonetheless, long-term follow-up studies in these group of young patients is warranted to gain better insights on the rates of HBeAg and HBsAg loss as well as the rates of seroconversion, in order to determine whether earlier treatment is genuinely associated with better treatment outcomes.

The emerging concept of “trained immunity” discussed above [57] also has significant therapeutic implications. Rather than being immature or tolerized, we should bear in mind that the immune system of neonates or even young children is already “trained” or “matured” following birth and is actually capable of responding immunologically with broad cross-protective responses towards viral antigens. In this context, therapies that reduce the expression of viral proteins may help to “release the brakes” on the immune system of these young patients, allowing it to be fully competent to achieve long-term suppression of HBV. On the other hand, our findings that young CHB patients in the “immunotolerant” disease phase can mount HBV-specific immune responses without inducing a full-blown pro-inflammatory reaction [69] suggest that therapeutic interventions aimed at enhancing HBV-specific immunity (e.g., vaccine therapy, checkpoint inhibitors) [12] are likely to be more effective in young CHB patients compared to adult patients. On a separate note, we should start to consider and evaluate CHB in adults in the context of an inflammatory, rather than a virus-induced disease, and as such, anti-inflammatory therapies designed to inhibit liver inflammation may turn out to be more effective in controlling HBV infection. The superior efficacy of anti-platelet therapy in blocking the development of HCC in HBV transgenic mice is one such example [85, 86].

## Conclusions and perspectives

Chronic HBV infection is a complex dynamic disease with an unpredictable course, where the timing and most appropriate treatments continue to be debated. As we have discussed, HBV transmission at birth does not inevitably lead to the induction of a generic immune tolerant state and the inability to mount HBV-specific adaptive immunity. In addition, the detection of HBV-specific T cell response and necroinflammatory activity and fibrosis on liver biopsy in young patients are at odds with the concept of complete tolerance. These findings underscore the limited value of characterizing the disease phase solely based on clinical and virological markers, and more importantly, raise doubts about the premise on which treatment decisions are made.

A better understanding of the concept of trained immunity and how HBV establishes a permissive state in the host may pave the way for better development of therapies that are targeted towards these groups of patients currently excluded for treatment. This could potentially result in expanding therapeutic options to more patients, including treating at a younger age and at a much earlier stage of disease. Having said this, the early phase of HBV infection, i.e., from HBV-exposed newborn infants to CHB-infected children, remains an area that needs further studies. The potential role and function of many other components of the immune response, including NK and NK-T cells that are present in abundance in the liver, should also be analyzed in relation to age [87]. Should more data be available to support our stance and that earlier treatment is advocated, careful consideration should be taken into account for pediatric patients, in whom the safety, efficacy, and adverse effect profiles of antivirals have not been well established compared to the adult population.
